# New Delhi metallo-β-lactamase-1-producing *Klebsiella pneumoniae* isolates in hospitalized patients in Kashan, Iran

**Published:** 2017-10

**Authors:** Farzaneh Firoozeh, Zeinab Mahluji, Ehsaneh Shams, Ahmad Khorshidi, Mohammad Zibaei

**Affiliations:** 1 Department of Microbiology and Immunology, School of Medicine, Kashan University of Medical Sciences, Kashan, Iran; 2 Department of Microbiology and Immunology, School of Medicine, Karaj University of Medical Sciences, Karaj, Iran; 3 Evidence-Based Phytotherapy and Complementary Medicine Research Center, Alborz University of Medical Sciences, Karaj, Iran; 4 Department of Parasitology and Mycology, School of Medicine, Alborz University of Medical Sciences, Karaj, Iran

**Keywords:** Carbapenemases, New Delhi metallo-ß-lactamase, Class 1 integron, *Klebsiella pneumoniae*

## Abstract

**Background and Objectives:**

New Delhi metallo-ß-lactamase (NDM) is a newly emerging metallo-ß-lactamases, which can destroy all β-lactams including carbapenems. Therefore, this study aimed at evaluating New Delhi metallo-ß-lactamase-1–production in clinical isolates of *Klebsiella pneumoniae* in Kashan, Iran.

**Materials and Methods:**

In a cross-sectional study, 181 *K. pneumoniae* isolates were collected from clinical samples of patients, who referred to Shahid Beheshi hospital in Kashan during November 2013 and October 2014. Antimicrobial susceptibility patterns were determined using disk diffusion method, according to CLSI guidelines. Metallo-ß-lactamase (MBL) production was identified among imipenem-resistant *K. pneumoniae* isolates using imipenem-EDTA double disk synergy test (EDTA-IMP DDST). PCR method and sequencing were used to detect integron Class 1 and *bla*_NDM-1_ gene. Statistical analyses were performed using SPSS software Version 16.

**Results:**

Of the 181 *K. pneumoniae* isolates, 36 (19.9 %) were imipenem-resistant strains. A total of 28 out of 36 (77.7%) imipenem-resistant *K. pneumoniae* isolates were identified as MBL producer strains. Also, 150 (82.9%) *K. pneumoniae* isolates carried *intI1* gene, and 20 (11.1%) *K. pneumoniae* isolates harbored *bla*_NDM-1_ gene.

**Conclusion:**

Our study revealed a high frequency of MBL production and the presence of *bla*_NDM-1_ among *K. pneumoniae* strains, especially among hospitalized patients, which is alarming. Moreover, the presence of Class 1 integrons in all multi-drug resistant *K. pneumoniae* isolates highlights the risk of rapid spread of the resistance genes, especially in clinical settings.

## INTRODUCTION

Carbapenems includes a Class of ß-lactams that can kill most bacteria and are recommended for treatment of infections caused by extended-spectrum β-lactamase (ESBL)-producing *Enterobacteriaceae*, mainly *K. pneumoniae* (1, 2). Carbapenem resistance due to the carbapenemase enzymes is one of the complicated health issues worldwide because carbapenemase producing clinical isolates simultaneously show resistance to carbapenems and to all other ß-lactam antibiotics (3). Metallo-ß-lactamases (MBLs) belong to Class B ß-lactamase and need zinc for their activity (4). MBLs have a wide spectrum of β-lactamase activity and can affect a wide range of β-lactam antibiotics including carbapenems (4). Bulk of the resistance genes including carbapenemases in *K. pneumoniae* are carried on Class 1 integrons. It has been documented that metallo-β-lactamases are associated with gene cassettes carried on integrons (5). Integrons as transferable genetic elements facilitate the transfer of resistance genes among different bacteria (6).

Among the newly identified metallo-β-lactamases, New Delhi metallo-ß-lactamase (NDM) is a recently described enzyme conferring resistance to all ß-lactams, especially carbapenems except monobactams (7). Since its first identification in New Delhi, India, in 2008, NDM has been reported by different countries around the world as an important health concern (8).

The *bla*_NDM-1_ gene, responsible for producing NDM in clinical isolates, is carried on transferable genetic elements, leading to rapid dissemination of these genes (7). There is limited data on NDM production and carriage of Class 1 integrons in clinical isolates of *K. pneumoniae* in our regain. Thus, the present study was conducted to identify the *bla*_NDM_ metallo-β-lactamase gene and the presence of Class 1 integrons in clinical isolates of *K. pneumoniae* in Kashan, Iran.

## MATERIALS AND METHODS

In this cross-sectional study, 181 clinical isolates of *K. pneumoniae* including urine (n = 124), respiratory tract samples (n = 43), blood (n = 5), wound (n = 6), cerebrospinal fluid (CSF) (n = 1), and catheter (n = 2) were isolated from patients, who referred to Shahid Beheshi hospital in Kashan during November 2013 to October 2014. The isolates were identified using standard microbiological methods (9). Antibiotic resistance profiles to the following antibiotics were determined using disk diffusion method: gentamicin (10 μg), ampicillin (30 μg), amoxicillin/clavulanic acid (20/10 μg), aztreonam (30 μg), cefotaxime (30 μg), ceftazidime (30 μg), cefoxitin (30 μg), cefteriaxone (30 μg), nalidixic acid (30 μg), ciprofloxacin (5 μg), trimethoprim-sulfamethoxazole (25 μg), ertapenem (10 μg) and imipenem (10 μg), (Mast, UK). Antibiotic susceptibility protocol was in accordance with the guidelines of Clinical and Laboratory Standards Institute (CLSI). The *Escherichia coli* (ATCC 25922) standard strains were used to perform quality control in susceptibility testing. To identify MBL-producing *K. pneumoniae* isolates, imipenem resistant isolates were studied using Imipenem-EDTA double disk synergy Test (EDTA-IMP DDST). Boiling method was used for DNA extraction of *K. pneumoniae* isolates. PCR amplification was used to detect *bla*_NDM-1_ gene using specific primers including NDM-F (5′-GGTTTGGCGATCTGGTTTTC-3′) and NDM-R (5′-CGGAATGGCTCATCACGATC-3′) (10). Strains that were confirmed to carry *bla*_NDM-1_ gene by sequencing were used as positive controls. The carriage of the Class 1 integrons was investigated by amplification of the *intI1* gene among MDR *K. pneumoniae* isolates using PCR assays. The primers used were as follow: *IntI1*-F (5′-TCTCGGGTAACATCAAGG-3′) and *IntI*-R (5′-AGGAGATCCGAAGACCTC-3′) (6). The PCR products were separated by electrophoresis on 1.2% agarose gel and seen in gel document system (Bio-Rad, UK). Some purified PCR products were sequenced (Bioneer, South Korea) and compared by online BLAST software (http://www.ncbi.nlm.nih.gov/BLAST/). SPSS software Version 16 (SPSS, Inc.) was used for statistical analyses. Differences were considered by the χ^2^ test, and *p*-values of less than 0.05 were determined as statistically significant.

## RESULTS

Of the 181 *K. pneumoniae* isolates of hospitalized patients, 78 (43.1%) were collected from male and 103 (56.9%) from female patients. The patients’ age ranged from 1 to 97, and their mean age was 50.36 years.

The antibiotic resistance profile is demonstrated in Table 1. Disk diffusion method revealed that 36 (19.9 %) of *K. pneumoniae* isolates were imipenem-resistant strains. MBL production was observed in 28 (77.7%) isolates of imipenem–resistant *K. pneumoniae* strains. A total of 150 (82.9%) *K. pneumoniae* isolates carried *intI1* gene and were Class 1 integron-positive isolates. Moreover, 20 (11.1%) *K. pneumoniae* isolates harbored *bla*_NDM-1_ gene and were recognized as NDM-producing isolates. The *bla*_NDM-1_ gene carriage was found in all hospital wards except Critial Care Unit (CCU), while the most frequent ward, which harbored the *bla*_NDM-1_ gene, was intensive care unit (ICU) (Table 2). The nucleotide sequencing of the PCR products of *bla*_NDM-1_ (the GenBank accession number: KP340793.1) and *intI1* genes were equal to those deposited in the GenBank.

**Table 1.** Antimicrobial resistance profile of *Klebsiella pneumoniae* isolates busing disk diffusion method

**Antibiotic**	**Isolates (n)%**		

	**Sensitive**	**Intermediate**	**Resistant**
Gentamicin	117 (64.6)	5 (2.8)	59 (32.6)
Ampicillin	5 (2.8)	3 (1.7)	173 (95.5)
Amoxicillin-clavulanic acid	63 (34.8)	26 (14.4)	92 (50.8)
Aztreonam	82 (45.3)	16 (8.8)	83 (45.9)
Cefotaxime	68 (37.6)	11 (6.1)	102 (56.3)
Ceftazidime	80 (44.2)	10 (5.5)	91 (50.3)
Cefoxitin	117 (64.6)	14 (7.8)	50 (27.6)
Ceftriaxone	78 (43.1)	6 (3.3)	97 (53.6)
Nalidixic acid	56 (30.9)	34 (18.8)	91 (50.3)
Ciprofloxacin	89 (49.2)	12 (6.6)	80 (44.2)
Trimethoprim-sulfamethoxazole	104 (57.5)	25 (13.8)	52 (28.7)
Imipenem	137 (75.7)	8 (4.4)	36 (19.9)
Ertapenem	130 (71.8)	12 (6.6)	39 (21.6)

**Table 2.** Association among age, sex, sample type, patient admission and *bla*_NDM-1_ gene in *Klebsiella pneumoniae* isolates

**Patient Characteristics**	***bla*_NDM-1_ Positive n= 20 (%)**	***bla*_NDM-1_ Negative n= 161 (%)**	***p*-value**
**Age**			
<50 years	13 (65)	128 (79.5)	0.4
≥50 years	7 (35)	33 (20.5)	
**Sex**			
Male	9 (45)	69 (42.9)	0.06
Female	11 (55)	92 (57.1)	
**Sample Type**			
Urine	10 (50)	114 (70.8.)	0.001
CSF	1 (5)	0 (0)	
Blood	0 (0)	5 (3.1)	
Catheters	2 (10)	0 (0)	
Wound	2 (10)	4 (2.5)	
Respiratory	5 (25)	38 (23.6)	
**Patient admission**			
Hospitalized	20 (100)	107 (66.5)	0.001≥
Out patients	0 (0)	54 (33.5)	
**Hospitalized ward**			
ICU	10 (50)	28 (17.4)	0.001≥
Infectious diseases	3 (15)	16 (9.9)	
Surgery	1 (5)	17 (10.6)	
maternity	2 (10)	9 (5.6)	
Pediatric	1 (5)	11 (6.9)	
Internal medicine	2 (10)	15 (9.3)	
Emergency	1 (5)	6 (3.7)	
CCU	0 (0)	5 (3.1)	
Outpatients	0 (0)	54 (33.5)	

The statistical analysis revealed a correlation (P < 0.05) among the hospitalized patients’ ward, sample type, and patient admission with NDM production (Table 2).

## DISCUSSION

Carbapenem resistant *K. pneumoniae* strains are increasing, and infections due to these strains are accompanied with higher mortality, length of hospitalization, and cost of treatment (11).

Our results revealed that 77.7% of imipenem–resistant *K. pneumoniae* strains produced MBL. Different frequencies of MBL production have been reported among *K. pneumoniae* strains (8). In contrast with our results, in a study conducted by Fazeli et al. in Isfahan, 10.2% of carbapenem-resistant *K. pneumoniae* isolates have been reported to be MBL producers (12). In another study in Greece, the prevalence of metallo-beta-lactamases in *K. pneumoniae* isolates from blood was 50% (13). The reason for the diverse prevalence of MBL production among *K. pneumonia* strains in different studies may be due to the use of different methods or different clinical samples. In addition, the discrepancy of phenotypic and genotypic features of bacterial isolates and factors such as cultural-economic status in diverse geographical areas could also be the reason. The results of our PCR assays demonstrated that 11.1% of *K. pneumoniae* isolates carried *bla*_NDM-1_ gene and produced NDM. In accordance with our findings, in a study conducted in Isfahan, 12% of carbapenem-resistant *K. pneumoniae* isolates were expressed New Delhi metallo-beta-lactamase (12), whereas, the prevalence of *bla*_NDM-1_ among *Enterobacteriaceae* in countries such as India and Kuwait has been reported to be higher (7, 14). Although the *bla*_NDM-1_ carrying *K. pneumoniae* strains are not very common in Iran, the results of this study is alarming. In most studies, NDM producer *K. pneumoniae* strains are resistant to most generally used antibiotics including β-lactams, β-lactamase inhibitors, fluoroquinolones, aminoglycosides and carbapenems (9, 15). The analysis of antibiotic resistance profiles of NDM producer *K. pneumoniae* strains in this study revealed that all NDM-producing *K. pneumoniae* isolates were multi-drug resistant strains, with resistance to almost all tested antibiotics; and this is in agreement with the results of other studies (12, 15). According to the literature, New Delhi metallo-beta-lactamase is a kind of beta-lactamase, which confers resistance to carbapenems and all β-lactam antibiotics except monobactams, such as aztreonam. In this study, in agreement with other reports, NDM-producing *K. pneumoniae* strains showed resistance to aztreonam along with other tested antibiotics (7, 12). The resistance to aztreonam in these *bla*_NDM-1_ positive *K. pneumoniae* strains may probably be due to other mechanism of resistance. The association between metallo-β-lactamase related genes including *bla*_NDM-1_ and mobile genetic elements, such as plasmid and integrons, has been documented (12, 13). We found that all multi-drug resistant *K. pneumoniae* isolates carried Class 1 integrons. The concomitance of NDM genes and mobile genetic elements, especially Class 1 integrons, facilitates their widespread propagation, which is a serious threat to the management of hospital-acquired infections. Furthermore, in this study, all NDM positive *K. pneumoniae* strains were isolated from hospitalized patients, and this is similar to reports by Jamal et al. indicating a nosocomial acquirement (7). Also, half of our NDM-producing *K. pneumoniae* strains were identified among hospitalized patients in ICU, where patients commonly have underlying diseases and experience long-term hospitalization and prolonged treatment with antibiotics, which facilitate the selection and spread of these resistant strains.

## CONCLUSION

This study revealed that the high frequency of MBL production and presence of *bla*_NDM-1_ among *K. pneumoniae* strains, especially among hospitalized patients, are highly alarming. Also, the presence of Class 1 integrons in all multi-drug resistant *K. pneumoniae* isolates highlights the risk of rapid spread of the resistance genes, especially in clinical settings.
